# Fracture in the Elderly Multidisciplinary Rehabilitation (FEMuR): a phase II randomised feasibility study of a multidisciplinary rehabilitation package following hip fracture

**DOI:** 10.1136/bmjopen-2016-012422

**Published:** 2016-10-05

**Authors:** Nefyn H Williams, Jessica L Roberts, Nafees Ud Din, Nicola Totton, Joanna M Charles, Claire A Hawkes, Val Morrison, Zoe Hoare, Michelle Williams, Aaron W Pritchard, Swapna Alexander, Andrew Lemmey, Robert T Woods, Catherine Sackley, Pip Logan, Rhiannon T Edwards, Clare Wilkinson

**Affiliations:** 1 School of Healthcare Sciences, Bangor University, Wrexham, UK; 2 Betsi Cadwaladr University Health Board, North Wales, UK; 3 Warwick Clinical Trials Unit, University of Warwick, Coventry, UK; 4 School of Psychology, Bangor University, Bangor, UK; 5 School of Sports, Health and Exercise Science, Bangor University, Bangor, UK; 6 School of Health and Social Care Research, King's College, London, UK; 7 School of Medicine, University of Nottingham, Nottingham, UK

**Keywords:** REHABILITATION MEDICINE, HEALTH ECONOMICS, QUALITATIVE RESEARCH, GERIATRIC MEDICINE

## Abstract

**Objective:**

To conduct a rigorous feasibility study for a future definitive parallel-group randomised controlled trial (RCT) and economic evaluation of an enhanced rehabilitation package for hip fracture.

**Setting:**

Recruitment from 3 acute hospitals in North Wales. Intervention delivery in the community.

**Participants:**

Older adults (aged ≥65) who received surgical treatment for hip fracture, lived independently prior to fracture, had mental capacity (assessed by clinical team) and received rehabilitation in the North Wales area.

**Intervention:**

Remote randomisation to usual care (control) or usual care+enhanced rehabilitation package (intervention), including six additional home-based physiotherapy sessions delivered by a physiotherapist or technical instructor, novel information workbook and goal-setting diary.

**Primary and secondary outcome measures:**

Primary: Barthel Activities of Daily Living (BADL). Secondary measures included Nottingham Extended Activities of Daily Living scale (NEADL), EQ-5D, ICECAP capability, a suite of self-efficacy, psychosocial and service-use measures and costs. Outcome measures were assessed at baseline and 3-month follow-up by blinded researchers.

**Results:**

62 participants were recruited, 61 randomised (control 32; intervention 29) and 49 (79%) completed 3-month follow-up. Minimal differences occurred between the 2 groups for most outcomes, including BADL (adjusted mean difference 0.5). The intervention group showed a medium-sized improvement in the NEADL relative to the control group, with an adjusted mean difference between groups of 3.0 (Cohen's d 0.63), and a trend for greater improvement in self-efficacy and mental health, but with small effect sizes. The mean cost of delivering the intervention was £231 per patient. There was a small relative improvement in quality-adjusted life year in the intervention group. No serious adverse events relating to the intervention were reported.

**Conclusions:**

The trial methods were feasible in terms of eligibility, recruitment and retention. The effectiveness and cost-effectiveness of the rehabilitation package should be tested in a phase III RCT.

**Trial registration number:**

ISRCTN22464643; Results.

**Strengths and limitations of this study**

- This study was designed to assess the feasibility of trial methods and intervention delivery and was therefore not powered to test the effectiveness or cost-effectiveness of the intervention.
- Different outcome measures were assessed to determine which would be the most suitable for a larger definitive randomised controlled trial.
- Comparative data were collected from an anonymised cohort, allowing comparison of characteristics with recruited participants and identification of differences.
- Ethical approval was granted only for recruitment of patients with mental capacity to consent, therefore excluding a large number of potential participants lacking capacity.

## Background

Proximal femoral fracture, more commonly referred to as hip fracture, is a common, major health problem in old age^1^ and as the population ages, the number of elderly people falling and fracturing their hips is projected to increase further.^2^ ^3^ Such fractures are strongly associated with decreased bone mineral density, increased age, prior fragility fracture, cognitive impairment, other health problems, undernutrition, frailty, poor physical functioning, vision problems and weight loss.^4^ Mortality is high with 14–58% dying within the following 12 months.^5^ ^6^ A review of the long-term disability associated with proximal femoral fracture found that 29% did not regain their level of functioning after 1 year in terms of restrictions of activities of daily living.^7^ Many who were living independently before their fracture lose their independence afterwards, which imposes a large cost burden on society amounting to about £2.3 billion a year in the UK.^8^

The National Institute for Health and Care Excellence (NICE) has issued guidelines for the management of hip fracture.^9^ As well as prompt surgical treatment and the management of associated medical needs, the guidelines recommend a programme of multidisciplinary rehabilitation. Such rehabilitation starts while in hospital during postoperative recovery, continues in the community following hospital discharge and has the potential to maximise recovery, enhance quality of life and maintain independence. While individual components of such programmes show promise, there is insufficient evidence of overall effectiveness or cost-effectiveness of an overall care pathway.^10–12^

The first phase of this research project developed a new community-based rehabilitation intervention^13^ within the Medical Research Council's (MRC) framework for complex intervention development.^14^ The aim of the second phase of the present study was to assess the feasibility of conducting a definitive randomised controlled trial (RCT) of this intervention and its acceptability through focus groups with therapists, patients and carers; and to conduct a concurrent economic evaluation.

## Study objectives

1. To assess the feasibility of a future definitive RCT by assessing eligibility, recruitment and retention rates, exploring the willingness of patient participants to be randomised and the willingness of patients and carers to complete process and outcome measures.
2. To assess the acceptability of the rehabilitation programme among patients, carers and clinicians, and to identify any adverse events.
3. To produce means and SDs of the quantitative measures, so that effect sizes can be calculated for planning the future RCT.
4. To explore the methodological issues for conducting an economic evaluation alongside a future RCT, and report exploratory economic analyses.

## Study design

Phase II comprised the second stage of the MRC framework^14^ and consisted of a randomised feasibility study, including focus groups of the multidisciplinary rehabilitation teams, hip fracture patients and their carers. An anonymous cohort study of all proximal femoral fracture patients was also conducted to assess the feasibility of recruiting a representative sample by comparing the recruited participants with the cohort population.

## Method

The protocol for this phase II study has been described elsewhere.^13^ The cohort consisted of an anonymised data set of all patients aged 65 years and over admitted to the three main acute hospitals of Betsi Cadwaladr University Health Board (BCUHB) in North Wales (Wrexham Maelor, Ysbyty Glan Clwyd and Ysbyty Gwynedd) with hip fracture during the first 6 months of the study period. They were followed up for 3 months. The following data were collected: the number admitted with proximal femoral fracture; the number who fulfilled the inclusion criteria for the randomised feasibility study; the number of deaths, serious complications and readmissions. Participants to the feasibility study were recruited on the orthopaedic wards while recovering from surgical treatment for proximal femoral fracture. We also recruited carers who were relatives or friends who provided help with activities of daily living for most days of the week. The specific inclusion criteria for hip fracture patients were given below.

### Inclusion criteria

- Age 65 years or older
- Recent proximal hip fracture
- Surgical repair by replacement arthroplasty or internal fixation
- Living in their own home prior to hip fracture
- Capacity to give informed consent, as assessed by the clinical team in the acute hospital. Patients with postoperative delirium were approached if this was resolved prior to discharge from the acute hospital.
- Living and receiving rehabilitation from the NHS in the area covered by BCUHB

### Exclusion criteria

- Living in residential or nursing homes prior to hip fracture
- Not able to understand Welsh or English

### Randomisation

Randomisation was performed remotely by researchers conducting baseline assessments, generating an email to physiotherapists delivering the intervention who assigned participants to the appropriate groups. Randomisation was by dynamic allocation^15^ to protect against subversion while ensuring that the trial maintained good balance to the allocation ratio of 1:1 within each stratification variable and across the trial. Participants were stratified by: (1) hospital and (2) gender. Stratification by hospital was necessary as each hospital has differing usual care pathways and, due to the geography of the area, different therapy teams delivered the intervention in different areas.

### Study interventions

An ‘enhanced’ rehabilitation intervention to improve patients' self-efficacy and increase the amount and quality of patients' practice of physical exercise and activities of daily living was compared with usual rehabilitation care. This intervention consisted of a patient-held information workbook and goal-setting diary (see online supplementary files 1 and 2) provided to the patient before or soon after discharge from the acute hospital. Six additional therapy sessions were delivered postdischarge by physiotherapists and technical instructors in the patient's place of residence or outpatients' department if required, with the timescale of delivery being decided by the therapist according to patient's individual needs. Session content was tailored to the individual and led by goals set by the patient in their initial intervention session, using the information workbook and supported by the therapist's clinical expertise of what was realistic and achievable for the patient. Patients and therapists reviewed and amended goals throughout the 3-month intervention period. The theory underpinning how the individual components interact to achieve desirable outcomes is discussed in detail elsewhere.^13^ Usual care was variable, consisting of multidisciplinary rehabilitation delivered by the acute hospital, community hospital and community services depending on patients' individual needs at different times during their recovery and on the availability and accessibility of services in different areas. Care pathways in this area did not include the provision of rehabilitation information leaflets on discharge from the acute hospital and any goal-setting activities were therapist-led.

10.1136/bmjopen-2016-012422.supp1 supplementary file

10.1136/bmjopen-2016-012422.supp2 supplementary file

### Outcome measures

Outcomes were collected in a variety of ways. Demographic data were collected from patients and their records. Researchers collected recruitment rates from their screening and recruitment records. Outcome measures (box 1) were completed by participants, assisted by a member of the research team who was blinded to treatment allocation, at baseline and at 3-month follow-up. Baseline measures were completed as soon as possible after surgery on the acute orthopaedic ward, inpatient rehabilitation ward or in the patient's home following discharge. Follow-up measures were completed at the patient's place of residence or in the physiotherapy department when attending for physical function tests, depending on the preference of the participant. Physical function was objectively assessed by the researcher at baseline using the grip strength test.^16^ At 3-month follow-up, a physiotherapist measured other objective tests of physical function in addition to the grip strength test, such as 30 s sit-to-stand,^17^ 8-foot get-up-and-go^18^ and the 50-foot walk test.^19^ These were performed in the physiotherapy gym, or in the patient's home if they were unable to travel. In addition, carers completed the Caregiver Strain Index.^20^

Box 1 **Patient-reported outcome measures**

Primary outcome measure

Barthel Index (BADL)^21^

Secondary outcome measures

Abbreviated Mental Test Score (AMTS)^22^

Nottingham Extended Activities of Daily Living (NEADL) scale^23^

Hospital Anxiety and Depression Scale (HADS)^24^

Visual Analogue Scale (VAS) for hip pain intensity^25^

General Self-Efficacy Scale (GSES)^26^

Falls Efficacy Scale-International (FES-I)^27^ ^28^

Self-efficacy for exercise scale^29^

Visual Analogue Scale-Fear of Falling (VAS-FoF)^30^

EuroQol EQ-5D (3L)^31^

ICEpop CAPability measure for Older people (ICECAP-O)^32^ ^33^

Client Service Receipt Inventory (CSRI).^34^

### Trial analysis

Feasibility was assessed by measuring eligibility, recruitment and retention rates.

To calculate a representative effect size for each of the outcome measures, either analysis of covariance, adjusting each patient's follow-up score with their baseline score, or a student's t-test, when no baseline data were available, was completed as outlined in the protocol.^13^ These methods established the 3-month follow-up outcome measurements of the two treatment groups. All analysis was completed on an intention-to-treat basis.

In order to estimate the SD of the primary outcome measure to be used in a power calculation for a future definitive RCT, we aimed for a sample size of 50 participants completing the study.^35^

An exploratory economic evaluation was conducted from a public sector multiagency perspective.^13^ Intervention costs for the enhanced rehabilitation programme were obtained from the local health board and applied to information received from the healthcare professionals delivering the intervention (eg, salary band, time spent with patient, costs of travel). Participant service use was obtained using the CSRI questionnaire,^34^ and was fully costed to obtain a mean cost per participant, per arm of health and social care service use, using national unit costs.^32^ ^36^ The EQ-5D (3L) was used to calculate quality-adjusted life years (QALYs) over the 3-month study period, using the area under the curve method;^32^ and ICECAP-O was used to calculate a capability index score for a cost consequences analysis.

### Focus groups

Detailed methods of sampling and analysis for focus groups are reported elsewhere.^13^ In brief, patients were asked at recruitment to the feasibility study if they were willing to take part in future focus groups. All those who expressed an interest were invited to take part at a later stage of their rehabilitation. Focus groups were arranged in areas local to the respondents. Clinical staff involved in the study were invited to take part in focus groups at the acute hospital they were associated with. Where clinical commitments prevented attendance, a one-to-one phone interview was offered as an alternative. Focus groups and interviews were led by a topic guide (see online supplementary files 3 and 4) recorded, transcribed and analysed using the framework approach to thematic analysis.^37^ Analysis was conducted by a study researcher, an independent researcher experienced in qualitative analysis, and overseen by the chief investigator to ensure credibility.

10.1136/bmjopen-2016-012422.supp3 supplementary file

10.1136/bmjopen-2016-012422.supp4 supplementary file

## Results

### Cohort

Four hundred proximal hip fracture patients were identified in the anonymised cohort study between June and November 2014. Comparison of the cohort population with participants in the randomised feasibility study demonstrated that proportions were similar with regard to gender, type of hip fracture and type of hip surgery (table 1). However, the cohort population was slightly older with a mean age difference of 4.5 years. The proportion admitted to each of the three acute hospitals was similar, but more than half of the feasibility study participants were recruited from Ysbyty Glan Clwyd. In the cohort, 58 patients (15%) had been readmitted to hospital at 3-month follow-up and there were 69 deaths (17%) (see online supplementary file 5, table 1). Eighty-nine (22%) patients in the cohort lacked mental capacity and the mean duration of acute hospital admission was 18.8 days (SD 19.4).

**Table 1:** Characteristics of patients in cohort and trial data sets

Characteristic	Trial data Mean (SD); range	Cohort data Mean (SD); range
Age	79.4 (7.6); 66–99	83.9 (7.7); 66–101
	**Trial data** **N (%)**	**Cohort data** **N (%)**
Gender		
Male	15 (25)	108 (27)
Female	46 (75)	292 (73)
Type of fracture		
Intracapsular	27 (44)	195 (49)
Extracapsular	20 (33)	126 (31)
Missing	14 (23)	79 (20)
Extracapsular fracture		
Pertrochanteric	1 (5)	2 (2)
Intertrochanteric	11 (55)	130 (82)
Subtrochanteric	2 (10)	13 (10)
Missing	6 (30)	8 (6)
Type of surgery		
Total hip arthroplasty	5 (8)	27 (6)
Hemiarthroplasty	29 (47)	159 (40)
Internal fixation	17 (28)	151 (38)
Intramedullary nailing	2 (3)	16 (4)
No surgery	0 (0)	29 (7)
Missing	8 (14)	18 (5)
Hospital		
Ysbyty Gwynedd	11 (18)	146 (37)
Ysbyty Glan Clwyd	34 (56)	123 (31)
Wrexham Maelor	16 (26)	131 (33)
Readmissions	2 (3)	58 (15)
Deaths	1 (2)	69 (17)

Accommodation	Before admission	After discharge	Before admission	After discharge
Private property	59 (97%)	44 (72%)	313 (78%)	104 (26%)
Sheltered accommodation	2 (3%)	3 (5%)	11 (3%)	1 (<1%)
Residential home	0 (0%)	1 (2%)	34 (8%)	12 (3%)
Nursing home	0 (0%)	0 (0%)	36 (9%)	29 (7%)
Community hospital	0 (0%)	1 (2%)	3 (1%)	224 (56%)
Other acute hospital	0 (0%)	0 (0%)	1 (<1%)	6 (1%)
Missing	0 (0%)	12 (19%)	2 (<1%)	24 (6%)

10.1136/bmjopen-2016-012422.supp5 supplementary file

### Feasibility study

#### Baseline

**Figure 1:**
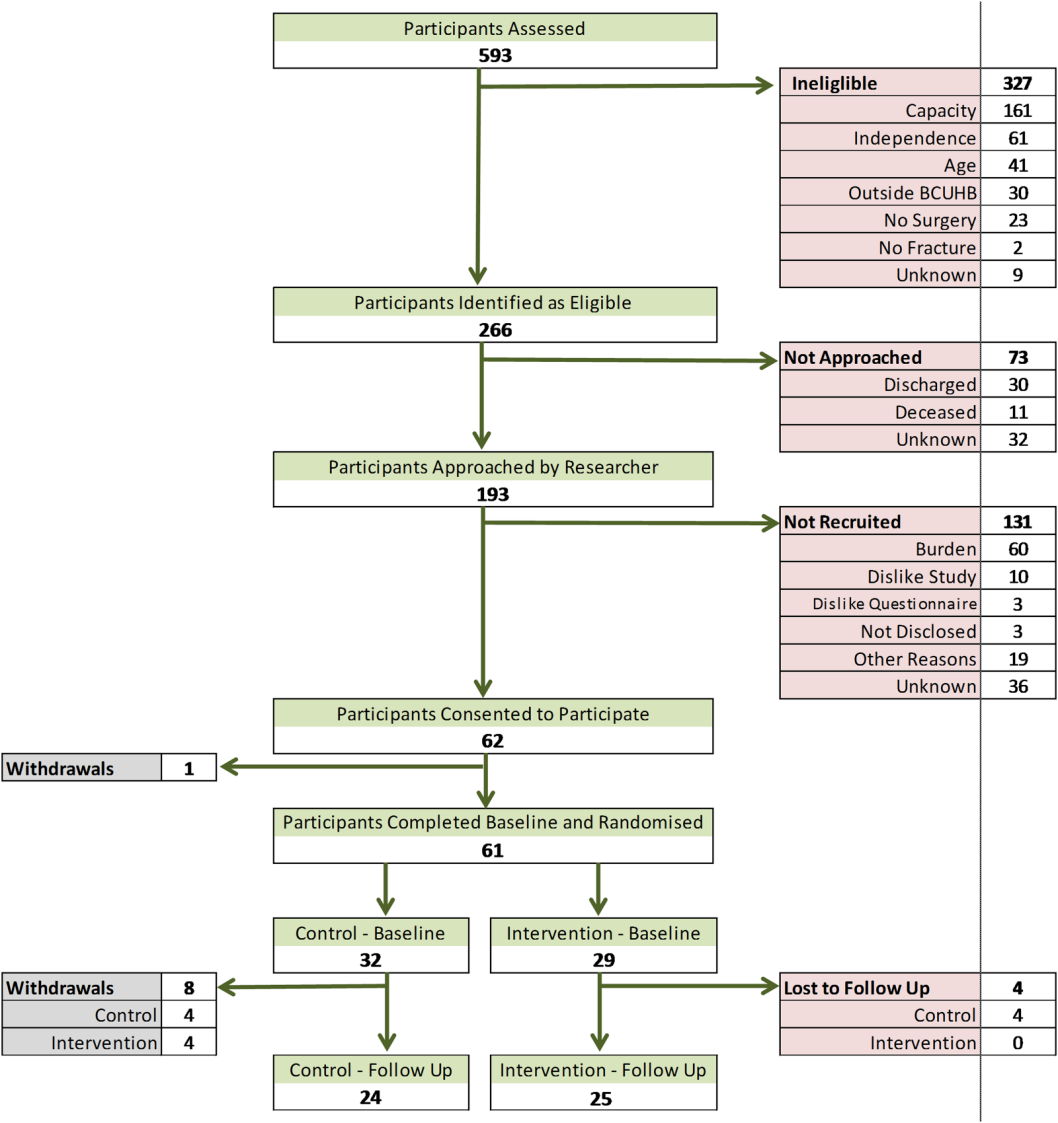
Feasibility study participant flow diagram.

Between June 2014 and March 2015, 593 patients with proximal femoral fracture were screened for eligibility, of which 266 (45%) were eligible (figure 1). The main reason for ineligibility was lack of mental capacity (49%). Out of those eligible, 193 (73%) were invited to participate and 62 (23% of the eligible population) agreed to participate. The main reason for non-participation was the perceived burden of the study. The majority of patients had two visits from researchers before they were recruited, and many requested a second visit to discuss the study after they had been discharged. From the recruited participants, 41 carers were identified, with 31 agreeing to participate (76%). The mean age of the intervention group was 2.9 years older than the control group, but the age of study participants ranged from 66 to 99 years (table 2). The proportions in the two groups were similar according to gender, living status, type of property, type of fracture, type of surgery and admitting hospital. After the hospital admission, there was a small discrepancy between those discharged directly to their place of usual residence (34% in the intervention group; 53% in the control group) and those sent to a community hospital for rehabilitation (52% in the intervention group; 22% in the control group). The baseline scores of the outcome measures and physical function tests were similar between the two groups (table 3). However, the Nottingham Extended Activities of Daily Living score was 2.4 points higher in the control group.

**Table 2:** Characteristics of patients split by treatment group

Characteristic	Overall mean (SD); range	Control mean (SD); range	Intervention mean (SD); range
Age	79.4 (7.6); 66–99	78.0 (8.3); 66–99	80.9 (6.6); 69–94
Abbreviated Mental Test Score (AMTS)	9.1 (1.3); 5–10	9.0 (1.2); 6–10	9.1 (1.3); 5–10
	**Overall** **N (%)**	**Control** **N (%)**	**Intervention** **N (%)**
Gender			
Male	15 (25)	9 (28)	6 (21)
Female	46 (75)	23 (72)	23 (79)
Usually lives			
Lives alone	31 (51)	16 (50)	15 (52)
Lives with others	30 (49)	16 (50)	14 (48)
Accommodation			
Owner-occupied property	48 (79)	24 (75)	24 (83)
Privately rented property	5 (8)	2 (6)	3 (10)
Housing association/local authority property	6 (10)	4 (13)	2 (7)
Sheltered accommodation	2 (3)	2 (6)	0 (0)
Type of fracture			
Intracapsular	27 (44)	16 (50)	11 (38)
Extracapsular	20 (33)	9 (28)	11 (38)
Not recorded/available	14 (23)	7 (22)	7 (24)
Type of surgery			
Total hip arthroplasty	5 (8)	4 (13)	1 (3)
Hemiarthroplasty	29 (47)	15 (47)	14 (48)
Internal fixation	17 (28)	7 (22)	10 (35)
Intramedullary nailing	2 (3)	2 (6)	0 (0)
Not recorded/available	8 (14)	4 (12)	4 (14)
Discharged directly to usual residence			
Yes	27 (44)	17 (53)	10 (34)
No	22 (36)	7 (22)	15 (52)
Missing	12 (20)	8 (25)	4 (14)
Hospital			
Ysbyty Gwynedd	11 (18)	6 (19)	5 (17)
Ysbyty Glan Clwyd	34 (56)	17 (53)	17 (59)
Wrexham Maelor	16 (26)	9 (28)	7 (24)

**Table 3:** Outcome measures including raw scores and adjusted mean differences from analysis of covariance analysis

	Baseline		Follow-up				
Outcome measure	Control group Mean (SD)	Intervention group Mean (SD)	Control group Mean (SD)	Intervention group Mean (SD)	Scale range	Adjusted mean difference between groups at follow-up (95% CI)	Effect size (95% CI)
Primary outcome measure							
Barthel index	17.8 (3.4) n=32	17.8 (2.4) n=27	17.7 (3.0) n=22	18.2 (2.9) n=21	0–20	0.5 (−0.5 to 1.6)	0.29 (−0.31 to 0.89)
Secondary outcome measures							
General Self-Efficacy Scale (GSES)	31.6 (5.6) n=31	33.6 (5.1) n=27	30.5 (7.8) n=19	33.7 (7.0) n=22	10–40	1.3 (−2.5 to 5.0)	0.20 (−0.42 to 0.81)
Hospital Anxiety and Depression Scale (HADS)	12.8 (9.0) n=31	11.0 (4.8) n=24	11.0 (8.2) n=22	8.7 (6.0) n=20	0–42	−1.2 (−4.8 to 2.6)	0.20 (−0.41 to 0.81)
** Nottingham Extended Activities of Daily Living (NEADL)**	**16.1 (5.5)** **n=29**	**13.7 (7.4)** **n=27**	**14.2 (5.7)** **n=20**	**15.8 (6.0)** **n=20**	**0–22**	**3.0 (−0.4 to 6.4)**	**0.63 (−0.01 to 1.26)**
Visual Analogue Scale (VAS) for hip pain intensity	4.2 (2.4) n=32	4.1 (2.3) n=28	3.4 (3.1) n=23	2.9 (2.9) n=25	0–10	−0.2 (−1.7 to 1.3)	0.00 (−0.58 to 0.58)
Carer outcome measure							
Carer strain index (CSI)	2.6 (3.1) n=12	3.5 (3.4) n=16	3.3 (3.7) n=8	2.7 (2.1) n=7	0–13	−1 (−5.1 to 3.0)	0.35 (−0.68 to 1.37)
Physical function tests							
Grip strength	21.0 (10.1) n=30	20.0 (7.2) n=26	23.4 (12.3) n=17	19.5 (8.3) n=12	Unlimited	1.2 (−1.7 to 4.2)	0.35 (−0.40 to 1.09)

Bold typeface denotes medium effect size.

#### Three-month follow-up

There were nine withdrawals, one before baseline and eight during the intervention (four from each group) (see online supplementary file 5, table 2). Four patients could not be contacted at follow-up, which gives a patient retention rate of 79%. Six of the carers withdrew during the study and seven were lost to follow-up. Eighteen completed the follow-up questionnaire, giving a carer retention rate of 44%. Nine adverse events were reported (three in intervention, six in control). Six of these were deemed as serious, including two readmissions (3%), one in the control and one in the intervention group, and one death (2%) in the control group, but none were related to the study (see online supplementary file 5, table 1). The differences between the two groups at 3-month follow-up are shown in tables 3 and 4. Cohen's d effect sizes have been calculated for each of the outcome measures. The main outcome measure the BADL, and most secondary outcomes had small effect sizes in favour of the intervention group. However, the NEADL showed a medium effect size, with a Cohen's d of 0.63, but a wide 95% CI (−0.01 to 1.26), also in favour of the intervention group. One of the physical function tests, the 50-foot walk test, was completed in a shorter time in the control group with a medium effect size (Cohen's d of 0.40), but again a wide 95% CI (−0.41 to 1.20). This seemed to be related to one outlier and so a sensitivity analysis was completed with this participant removed. This changed the effect size to 0.02 (95% CI of −0.80 to 0.84). General self-efficacy and self-efficacy for exercise had the lowest rate of completion at follow-up; with patients expressing confusion to researchers conducting the interviews about how to complete these measures (see online supplementary file 5, table 3).

**Table 4:** Outcome measures including raw scores and effect size

	Follow-up				
Outcome measure	Control group Mean (SD)	Intervention group Mean (SD)	Mean difference between groups at follow-up (95% CI)	Scale range	Effect size (95% CI)
Secondary outcome measures					
Falls Efficacy Scale-International (FES-I)	36.2 (14.9) n=17	32.0 (12.2) n=20	−4.2 (−13.2 to 4.8)	16–64	−0.31 (−0.96 to 0.35)
Self-Efficacy for Exercise Scale (SEE)	49.9 (21.7) n=18	58.2 (17.8) n=18	8.3 (−5.2 to 21.7)	0–80	0.42 (−0.25 to 1.08)
Visual Analogue Score-fear of falling (VAS-FOF)	4.8 (2.9) n=24	5.0 (2.5) n=23	0.2 (−1.4 to 1.8)	0–10	0.07 (−0.50 to 0.64)
Physical function tests					
Eight-Foot Get-Up-and-Go Test	13.6 (6.1) n=15	12.9 (6.0) n=12	0.6 (−5.4 to 4.2)	Unlimited	0.12 (−0.64 to 0.88)
** Fifty-Foot Walk Test**	**19.3 (6.7)** **n=12**	**31.5 (42.3)** **n=12**	**12.2 (−13.5 to 37.8)**	**Unlimited**	**0.40 (−0.41 to 1.20)**
Thirty-second Sit To Stand	11.0 (3.6) n=11	10.1 (3.9) n=10	−0.9 (−4.3 to 2.5)	Unlimited	0.24 (−0.62 to 1.10)

Bold typeface denotes medium effect size.

### Economic analysis

At 3-month follow-up, 49 sets of data were available for analysis. We excluded 6 because of missing data, leaving 43 complete cases for the economic (cost-consequence) analysis (intervention n=21, control n=22). The economic sample represented 72% of the main clinical sample, with similar baseline characteristics (eg, gender, mean age, living status, type of property, type of fracture and type of surgery). The control group had lower baseline scores for the EQ-5D^36^ index and VAS, and ICECAP O^32^ capability index than the intervention group. However, both groups had improved scores at follow-up (table 5). The difference in QALYs between the two groups was 0.02 (1000 bootstrapped 95% CI −0.02 to 0.06), equating to 8 days gained in the intervention group. The difference in capability indices between the two groups was zero (1000 bootstrapped 95% CI −0.11 to 0.22) (table 5). It cost a total of £6711 (£231 per person) to deliver the intervention. At baseline, participants in the control group accessed more services than the intervention group. GPs were the health professional the participants were most likely to see, with practice nurses their second highest accessed healthcare professional. During the 3 months prior to baseline costs to primary care services were minimal, with the costs of secondary (hospital) services accounting for the majority of total service costs (98%). During the 3-month prior to follow-up, the control and intervention groups accessed primary care services and social care services, predominantly. There were high levels of polypharmacy in the economic sample, on average, taking more than five medicines at the time of completing the CSRI questionnaire.^34^ Service use costs were mainly accrued by secondary (hospital) services (96%), as patients underwent surgery and required time in hospital to recover. Fifty-one per cent (n=22) of the sample reported longer than average inpatient stays postsurgery. At follow-up, the intervention group reported an average length of stay of 15.2 days, and the control group 10.5 days in an inpatient orthopaedic trauma ward, compared with the NHS average length of stay of 14.3 days. Also at follow-up, the intervention group reported an average length of stay at a rehabilitation inpatient ward of 12.1 days, and the control group 18.8 days, compared with the NHS average of 11.6 days. Longer inpatient stays were reported by 67% (n=14) of the intervention group and 36% (n=8) of the control group. The mean total service use costs at follow-up (including intervention costs) in the intervention group were £43 999 (1000 bootstrapped 95% CI £4027 to £88 818) higher than the control group (table 5). This was due to a larger proportion of the intervention group having prolonged inpatient stays compared with the control group.

**Table 5:** Economic outcome measures and costs*

	Intervention group (n=21)		Control group (n=22)		
Outcome measures and costs	Baseline Mean (SD)	3 month follow-up Mean (SD)	Baseline Mean (SD)	3 month follow-up Mean (SD)	Difference between groups (1000 bootstrapped 95% CI)
EQ-5D (3L) utility index score	0.50 (0.26)	0.37 (0.43)	0.66 (0.27)	0.60 (0.27)	–
EQ-5D (3L) VAS	64.43 (16.37)	55.14 (25.72)	71.10 (17.89)	68.55 (18.44)	
ICECAP O capability index	0.82 (0.11)	0.75 (0.21)	0.84 (0.13)	0.78 (0.19)	
Mean QALY over 3 months (1000 bootstrapped 95% CI)	0.15 (0.12 to 0.17)		0.12 (0.09 to 0.15)		0.02 (−0.02 to 0.06)
Mean change in ICECAP O capability index over 3 months (1000 bootstrapped 95% CI)	−0.03 (−0.08 to −0.03)		−0.03 (−0.12 to 0.07)		0 (−0.11 to 0.22)
Mean total service use costs at follow-up including cost of intervention (1000 bootstrapped 95% CI)	£149 243 (£119 376 to £186 036)		£105 244 (£78 935 to £132 971)		£43 999 (£4027 to £88 818)

*Mean EQ-5D utility indices, EQ-5D VAS scores, ICECAP O indices, QALYs and change in ICECAP O capability indices rounded to two decimal places; total service use costs rounded to the nearest £.

EQ-5D (3L), EuroQol-5 Dimensions (3 Levels); ICECAP O, ICEpop CAPability measure for Older people; QALY, quality-adjusted life year.

### Focus groups

Four focus groups were conducted with 13 patients and 6 carers and 2 focus groups with 7 healthcare professionals involved in the intervention (table 6). Because of the geographical spread of participants and clinical commitments of therapists in Gwynedd and Anglesey, it was not possible to conduct focus groups in this area, but a control group participant was able to attend a focus group in central region and one physiotherapist and two technical instructors participated in individual telephone interviews. The study was acceptable to patients, carers and therapists and the intervention was viewed positively. The most useful component according to participants was the extra sessions that they received. The goal-setting diary and workbook were seen as useful supporting documents by the majority of intervention group participants. Other findings were that in the context of variable usual rehabilitation care, which led to uncertainty among patients and carers about what to expect, the role of the therapist was extremely important in managing patients' expectations. Lack of information, particularly in the usual care group, led to unrealistic expectations in patients, with anger and frustration at their perceived lack of progress, which was addressed to some degree by the study intervention documents. An important part of the therapist's role was to reassure patients that they were progressing normally, and to give patients confidence that they could perform their physical activities safely and to counteract the fear of falling. This was particularly important to patients at the beginning of their rehabilitation, but therapists, particularly fully qualified physiotherapists, often overlooked its importance. Technical instructors appeared to be more aware of their role in psychological support of the patient, in addition to physical rehabilitation. Patients valued individualised care and support, which included the recognition of patients' unique rehabilitation needs, tailoring of care to suit these needs, and personalised goal-setting as a motivational tool. These activities were well supported by the workbook and the goal-setting diary and therapists commented that these supporting documents may be of particular use to those with cognitive impairment. Regular home visits with intervention therapists provided consistency and allowed a relationship to build between patient and rehabilitation therapist, which was important for patient engagement.

**Table 6:** Focus group participant details

Participant type	Location	Attendees
Patient and carers in the control group	East	Patient, F, 69 years, 14 months postdischarge, discharged directly home and Carer, M Patient, F, 80 years, 14 months postfracture, discharged directly home and Carer, M Patient, M, 67 years, 7 months postdischarge, discharged directly home (n=5)
Patient and carers in the control group	Central	Patient, F, 67 years, 11 months postfracture, discharged directly home Patient, M, 67 years, 12 months postfracture, discharged directly home and Carer, F Patient, F, 83 years, 6 months postfracture, inpatient rehabilitation prior to discharge home Patient, F, 75 years, 12 months postfracture, inpatient rehabilitation prior to discharge home (n=5)
Patient and carers in the intervention group	East	Patient, F, 86 years, 5 months postfracture, discharged directly home Patient, F, 86 years, 8 months postfracture, inpatient rehabilitation prior to discharge home Patient, F, 69 years, 14 months postfracture, discharged directly home (n=3)
Patient and carers in the intervention group	Central	Patient, F, 70 years, 7 months postfracture, discharged directly home Patient, M, 81 years, 6 months postfracture, discharged directly home and Carer, F Patient, M, 74 years, 5 months postfracture, discharged directly home and Carer, F Patient, F, 80 years, 7 months postfracture, inpatient rehabilitation prior to discharge home and Carer, M (n=7)
Healthcare professionals	East	Clinical specialist physiotherapist, two orthopaedic physiotherapists, physiotherapy technical instructor (n=4)
Healthcare professionals	Central	Orthopaedic acute physiotherapist, rotational physiotherapist, physiotherapy technical instructor (n=3)
Healthcare professionals	West	One-to-one phone interviews were conducted with an acute orthopaedic physiotherapist and two technical instructors

## Discussion

### Summary of main findings

Participants recruited to the cohort and feasibility study were similar in terms of gender, type of hip fracture and type of surgery, but the feasibility study participants were younger and less likely to die or be readmitted to hospital during the 3-month follow-up period. A mean AMTS score of 9.1 also indicates low levels of cognitive impairment. Recruitment and retention rates were acceptable, although the recruitment process was challenging due to the need to approach patients soon after surgery following a traumatic injury. The majority of patients required multiple visits prior to consent. The trial methods were feasible in terms of randomisation and outcome measure collection. The new rehabilitation intervention was acceptable to patients and clinicians. The intervention group showed a moderate improvement in their ability to perform activities of daily living as well as small improvements in self-efficacy and mental health. Paradoxically, the control group showed a moderate improvement in the 50-foot walk test, but this was shown to be down to an outlier. The NEADL was more responsive than the BADL and the most responsive measure of self-efficacy was the FES-I.

The economic evaluation found that the intervention cost £231 per person to deliver. There was a small relative improvement in QALY in the intervention group, albeit with a wide CI. There was an equal drop in ICECAP-O capability indices in both groups. Service use costs were greater in the intervention group, due to longer inpatient stays unrelated to the rehabilitation intervention.

The focus group findings were that in the context of variable usual rehabilitation care, the role of the therapist was extremely important in managing patients' needs and expectations. This was especially so at the beginning of rehabilitation, for giving permission about what physical activity was safe to do. Regular home visits allowed a relationship to build between patient and rehabilitation therapist, which was important for patient engagement. Patients valued the use of tailored care and personal goal setting as a motivational tool. These activities were well supported by the workbook and the goal-setting diary.

### Strengths and weaknesses

For reasons of patient confidentiality, the cohort used anonymised data, so it was not possible to match participants in the cohort with those in the feasibility study. The numbers suggest that the feasibility study identified 81% of those in the cohort. It is not known how the 19% who were not identified in the feasibility study differed, nor is it known why they were identified in the cohort but not in the feasibility study.

This was a single-centre feasibility study conducted in one local health board at its three acute hospital sites in North Wales. It was able to assess the feasibility of trial methods in terms of recruitment, randomisation and outcome measurement. The NHS REC did not permit us to recruit participants who lacked mental capacity, which has implications for the generalisability of our findings in this important group. A process evaluation was performed as part of the feasibility study and will be reported separately.

We were also able to assess different outcome measures, to determine which would be the most suitable for a larger definitive RCT. As a younger, healthier subpopulation of the cohort was recruited to the feasibility study, baseline scores for the BADL were high, causing a ceiling effect in this measure. The NEADL was more responsive than the BADL for measuring the ability to perform activities of daily living in this population. The self-efficacy scales for falls and for exercise were more responsive than the general self-efficacy scale, but researchers reported that many participants struggled to understand the exercise self-efficacy scale, so the falls self-efficacy scale (FES-I) seems the most appropriate measure of self-efficacy for the main definitive RCT. The most appropriate health economic outcome measure was EQ-5D. The physical function test with a medium effect size, ‘50-foot walk’ test, showed better function in the control than the intervention group. There were several possible explanations for this anomalous result. An outlier was identified which contributed to the apparent difference and in addition, physical function tests were performed on average 3 weeks later in the control group, so participants had longer to recover from surgery. Because of the nature of the 50-foot walk test, no adjustment could be made for patients' function at baseline. The only physical function test to have this adjustment was grip strength and here the difference between the groups at follow-up was reduced by including the baseline score as a covariate. It was also only possible to include results for participants who were able to complete the test without using any adaptations such as the use of a walking aid. While the use of cost-utility or cost-effectiveness analyses have been recommended by NICE (2011) as needed in the area of proximal femoral fracture, they were not appropriate for use in this feasibility study as it was not powered to demonstrate effectiveness. However, the cost-consequence analysis used is championed as a method particularly relevant to economic evaluations alongside public health interventions.^38–41^

While the content of intervention and usual care sessions may have contained similar exercise activities, care pathways in this area did not use patient-led goal-setting or provide written information on what to expect during recovery. In addition, the provision of usual care sessions was variable, while the intervention offered continuity and a definite number of sessions. These were major components of the intervention, which were only available to participants randomised to that group. While it is possible that other participants may have viewed information materials if they were used in group sessions, the one-to-one aspect of the delivery minimised the possibility of intervention contamination.

### Comparison with previous literature

Although there have been other studies of rehabilitation interventions that combined the promotion of physical exercise and practice of activities of daily living with psychological interventions designed to tackle self-efficacy and fear of falling,^29^ there have been none set in the UK NHS. While we observed fewer deaths and readmissions in the feasibility study population compared with our cohort population, the recruitment of younger, healthier patients to a physical activity or exercise intervention study was not surprising and has been reported previously.^42^ The overall recruitment rate of eligible patients was 23% and the main reason for non-recruitment was perceived study burden. Challenges in recruitment into trials, particularly exercise-based studies, are well documented.^43^ ^44^ The intervention applied in this feasibility trial used personalised goal setting and diaries to provide a record of progress during rehabilitation, using a similar ethos to two earlier studies^45^ ^46^ of patient-centred approaches to rehabilitation. One found that an integrated care pathway with a focus on motivation for rehabilitation and early ambulation was less costly and more effective than the usual care pathway.^45^ The other found that an accelerated rehabilitation intervention was more cost-effective than usual care.^46^ Goal setting and supporting patient's self-efficacy was seen to be important in helping patients engage with their rehabilitation. Another study on patient empowerment^47^ also found that empowered patients were more likely to benefit from their rehabilitation and return to previous living.

### Implications for future practice and research

This intervention should be tested in a definitive phase III RCT. It would be advantageous to include those who lack mental capacity as this would improve generalisability of trial results and increase the pool of potential participants. Although we were not able to test the feasibility of recruiting these patients, feedback from healthcare professionals highlighted the potential benefit of the intervention to those lacking capacity. Owing to the observed ceiling effect in the BADL in our recruited population, the primary outcome measure should be the NEADL for effectiveness and EQ-5D for the economic evaluation. The FES-I should be used to measure self-efficacy. The adjusted mean difference in NEADL between groups in this feasibility study was three, which is considered a clinically significant change. Others have suggested that the minimum clinically significant difference is 2.4 and so this has been used within the sample size calculation for a future study.^48^ Based on a t-test with α of 5% and 90% power to detect a difference of 2.4 (SD=5.86), 254 participants would be required to complete the trial over both treatment groups. When considering the 79% retention rate, a full trial of similar design would need to recruit 322 participants.

## Conclusions

While recruitment was challenging, we achieved acceptable recruitment and retention rate. Screening methods successfully identified 81% of patients with hip fracture, but the feasibility study recruited a younger sample with fewer complications compared with the anonymised cohort. The intervention was acceptable to patients, carers and healthcare professionals, and the intervention was viewed positively. The trial methods were feasible, including the collection of costs and outcome data for a future economic evaluation. Baseline scores in the intervention and control groups of the feasibility study were similar, but there was imbalance in the NEADL, which had a medium effect size while most outcome measures had a small effect size in favour of the intervention. Owing to the ceiling effect observed in the BADL and the greater responsiveness of the NEADL, this should be the primary outcome measure of a future definitive RCT.
