# Supplementary material for: Fracture in the Elderly Multidisciplinary Rehabilitation (FEMuR): a phase II randomised feasibility study of a multidisciplinary rehabilitation package following hip fracture
Source: BMJ Open. 2016 Oct 5;6(10):e012422. doi: 10.1136/bmjopen-2016-012422 (PMC5073533; doi:10.1136/bmjopen-2016-012422)
Supplement: supplementary file [file bmjopen-2016-012422supp1.pdf]

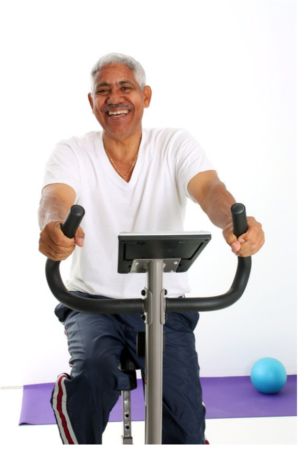
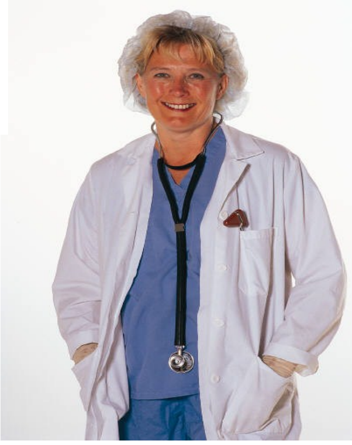
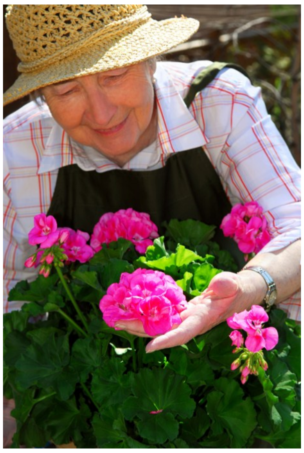

Hip Fracture Rehabilitation Workbook

A supported self-management programme
for people who have had a hip fracture

Bwrdd Iechyd Prifysgol Betsi Cadwaladr University Health Board

PRIFYSGOL BANGOR UNIVERSITY

NISCHR
Sefydliad Cenedlaethol ar gyfer Ymchwil Gofal Cymdeithasol ac Iechyd | National Institute for Social Care and Health Research

Funded by the National Institute for Health Research, Health Technology Assessment (NIHR HTA) the workbook was

developed by Jessica Roberts, Claire Hawkes, Nefyn Williams, Michelle Williams, Val Morrison, Andrew Lemmey, Catherine Sackley, Pippa Logan,

Swapna Alexander, Glynne Andrew and Nafees Din.

Contents

This workbook is made up of 3 main parts. There is also a space to monitor your progress and a list of useful contacts at the end. Please use the workbook with the healthcare professionals who are assisting with your rehabilitation

Introduction 4 - 9

Information about hip fracture and why it can effect you the way it does. It will also tell you how this workbook can help your recovery.

What to Expect 10 - 23

Information about what happens after surgery and practical things you may have questions about

Recovery 24 - 34

Information and activities to help your recovery.

Workbook Progress 35

Useful Contacts 36 - 37

Introduction

What is a Hip Fracture?

About This Workbook

What is a hip fracture?

A hip fracture is a crack or break in the top of the thigh bone (femur) close to the hip joint. It is called a proximal femoral fracture or fractured neck of femur.

The hip joint is a ball and socket joint. The ball (head) at the end of your thigh bone fits into the socket in your pelvic bone.

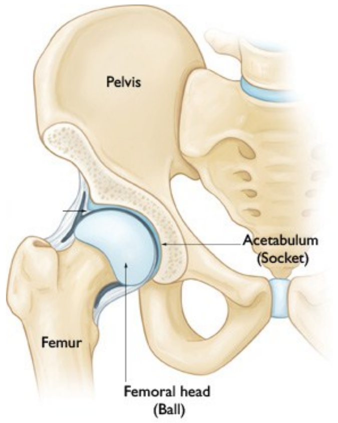

http://www.piersbarrymd.com/hip

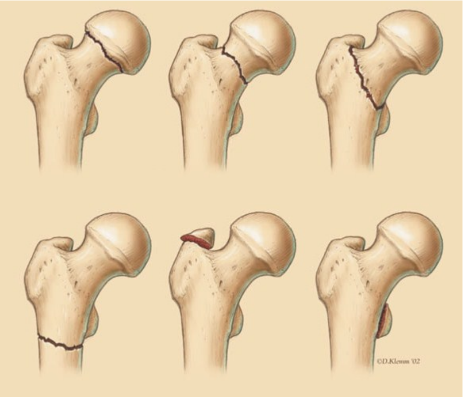

http://www.aafp.org/afp/2003/0201/p537.html

There are different types of hip fracture, and this can affect the treatment you will need. A fracture can happen in the part of the bone that sits inside the socket (intracapsular), or the part of the bone that is outside the socket (extracapsular).

Surgery is normally needed to treat a hip fracture because it cannot be put in a cast like other bones, so it has to be held in place from the inside whilst it heals. Your fracture may have been fixed using screws and plates to hold the bones in place, or part or all of the hip may have been replaced.

The type of surgery that you had will depend on the type of fracture that you have, how active you were before your fracture and the condition of the bone and joint. The type of surgery you have may also affect how well you can carry out certain activities after your operation.

Now that the surgery part of your treatment is completed you will need time and rehabilitation to help you recover. This workbook is designed to guide you through this recovery process.

Hip Fracture Workbook

You are receiving this workbook and goal setting diary because you have agreed to take part in a study that aims to improve the rehabilitation of people who have had surgery to treat a hip fracture. It will be used alongside your usual rehabilitation.

We hope that the workbook will provide you with information and help you take control over your recovery. People who feel in control and are actively involved in their rehabilitation may do better emotionally and physically.

This workbook will assist you to do this by:

- Providing you with information to help you and your family understand what to expect in your recovery and know what to do to help it.
- Helping you set realistic and achievable goals and give you a chance to look at how you are progressing
- Providing a space to write down how you might be feeling about your hip fracture and recovery and reflect on this
- Helping you identify challenges and find ways of coping with them
- Directing you to support services you can contact

This workbook gives you information and activities to help you make progress. You don't have to start at the beginning and work to the end. You can read the sections that feel most relevant to you and use what you find helpful. We hope that you will use this workbook and goal setting diary and that it will help you to know what you can do yourself, and what help is available when you need it.

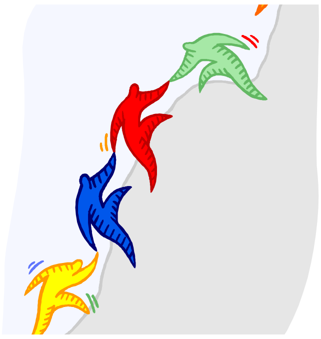

Remember recovery is not always obvious. Let some time pass before you look back at your progress.

Who is this workbook for?

- **Patients**

People of all ages break their hip. This workbook is for all of you but because everyone has individual needs your rehabilitation will be unique.

Your rehabilitation will be affected your health, fitness, mobility and care needs before you broke your hip. It will also be affected by how much you are able to put into your rehabilitation. We have provided many examples throughout this book but not all will be relevant to you in your situation. It is important to discuss with your health care staff what you would like to achieve, what is realistic in your current situation and how they can help you get there. Rehabilitation lasts a long time and there may be ups and downs along the way. We hope this workbook will help you get as much as possible out of your rehabilitation.

- **Family and friends**

You may be reading this book because you are a family member, friend or carer of someone who has had a hip fracture. If this is the case, we hope you will be able to use this book with your relative or friend and their healthcare staff to help them with their rehabilitation. We hope to increase your understanding of what to expect and how you may be able to support them in their recovery.

If your friend or family member has impaired memory or other conditions that make it difficult for them to use the workbook, you may want to read the book yourself. You may find it helpful to talk to the person who has broken their hip about what is in the workbook and explain things you think they might find helpful.

Quiz #1

Tick the box to indicate if you think the statement is true or false.

	True	False
It can help your recovery if you feel more in control of your rehabilitation	☐	☐
Your recovery won't be affected by how fit and healthy you were before your fracture	☐	☐
Having more information about hip fracture may help you feel more in control	☐	☐
Everyone's recovery is the same	☐	☐

Quiz #1: answers and discussion

"It can help your recovery if you feel more in control of your rehabilitation"

True - Taking control over your recovery has been shown to help you both physically and emotionally. You can feel more in control by getting more information about your hip fracture and your rehabilitation. It may also help to decide what is important to your own recovery and to set personal goals.

"Your recovery won't be affected by how fit and healthy you were before your fracture"

False - Your general health and fitness before your fracture will have an impact on your recovery, particularly if you have conditions that affect your mobility and ability to exercise. This is important to remember when setting realistic and achievable goals and keeping yourself motivated during your recovery.

"Having more information about hip fracture may help you feel more in control"

True - Having control over your recovery involves being armed with information and using it to work out what help you may need. You can get information from reading the workbook and talking to your healthcare team.

"Everyone's recovery is the same"

False - There are lots of factors which affect how well you recover and how quickly. It is a very personal process and your experience will be unique. The exercises you are given and the treatment you receive will be tailored by your healthcare team to suit your specific needs.

What to Expect

What Happens After Surgery?

My Hip Fracture Story

Things to Think About

Leaving Hospital

Other People's Experiences

The Role of the Health and Social Care Team

What happens after surgery?

After you have had surgery to fix your hip fracture you will be quite weak and will need time and help to recover.

Each person who has had a hip fracture will have a different experience and will be able to do different things. Because of this there is no specific pathway for recovery. You will be assessed by your healthcare team who will manage your day-to-day plan of care and rehabilitation activities. This team may include:

- Physiotherapists (PTs) and PT assistants
- Occupational therapists (OTs) and OT assistants
- Nurses
- Social workers

To have a successful recovery you will need to build up your strength again, and for this you will need to do physical exercises and activities. There is more information about physical exercise and its role in your rehabilitation in the recovery section of this workbook which starts on page 24.

You are not alone in your recovery, your healthcare team will help you plan your rehabilitation and will guide you as to which physical activities are appropriate for you. They will also help you set your own goals and make a plan for how you can achieve them. You can ask your healthcare team for feedback on your progress and any other questions you might have.

The team will help you in different ways depending on what you most need help with and where you are in your recovery. You can find more detailed information about how they will be involved in your rehabilitation on page 20.

My hip fracture story

On page 18 you will find some stories about other people who have had a hip fracture and their experience of it. You might like to write some notes about your own story and think about what you might like to achieve in your recovery and the things that might help you. You can use this space to make notes about things you might want to discuss with your healthcare team or your family and friends.

How did your hip fracture happen?

____

____

What was it like in the hospital?

____

____

What challenges have you overcome in your recovery so far?

____

____

What is the most important aspect of recovery for you?

____

____

____

Is there anything specific you are worried about or would like help with?

____

____

____

Things to think about

Whilst hip fracture is relatively common, the surgery that is needed to fix it is a major operation and you will feel very weak afterwards. You may be in pain, though this should be managed by your healthcare team. It is important to speak to the medical staff if you are in pain as this will make it more difficult to move around and care for yourself. It is common to feel emotional or worried as a result.

There are likely to be days when you feel especially tired or low and this may continue after you have gone home. This is not unusual but it is important to find ways to improve this. Speaking to your friends, family, healthcare professionals and using this workbook may help. Try not to feel discouraged when you have a bad day or if you can't do something. Come back to it later or try again tomorrow.

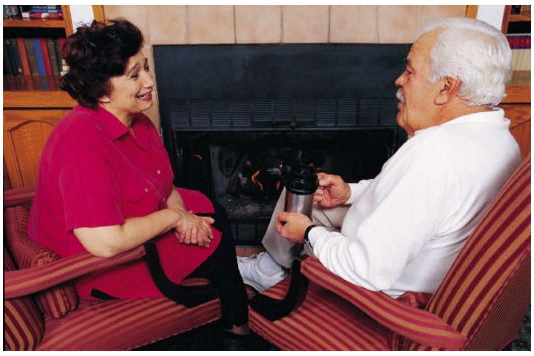

On the next few pages there are a number of common things that people recovering from a hip fracture might worry about, and some advice on how to overcome these challenges.

You may have other questions about your recovery, like how to care for your wound after surgery, or other medical conditions which you feel are having an impact on your recovery. This workbook focuses on rehabilitation, and if you need more information about any other aspects of your recovery you should speak to your healthcare team or GP. There are also contact numbers at the back of this book for a range of services which you may find useful.

“When will I be able to leave the hospital?”

One of the biggest things people may worry about following surgery for hip fracture is when they will get home and how they will cope. The time you spend in hospital depends on your individual circumstances. Around half of people are discharged back to their own home after around 7 or 10 days in the acute hospital. Going back to your own home raises some specific challenges so you might like to look at the information on pages 15 & 16 if this is relevant to you. If you need more care or rehabilitation there is information about this on page 17.

“I am scared I might fall again.”

It is normal to feel anxious at first, but doing the exercises your physiotherapist has given you will help improve your strength and balance, making you less likely to fall again. Take your time when you are moving around and try to make sure the floor is free from clutter. Wear supportive shoes and try to avoid wearing slippery socks or stockings. There is more advice about reducing your risk of falling on page 32.

“**“I’m worried I will be a burden to my family.”**

Whilst you might need extra help after your surgery, your family will probably be happy to be able to give you practical help. It is important for your recovery that you ask for help when you need it and don’t push yourself too hard. If you are worried about the strain on your family you can put them in touch with the Welsh Carer Network, details can be found on the contact page of this book.

“**What if I dislocate my new hip?”**

Some people who have had a hip replacement to treat their fracture worry about dislocating the new hip, especially when doing rehabilitation exercises. You will have been given plenty of information about how to avoid this, such as sitting properly and trying not to turn suddenly. Whilst taking care of your new hip is important you should still try and stay active and do what you are able to. The healthcare team will only give you exercises that are suitable for you to carry out safely so it is important to follow their advice and ask if you have any concerns.

Moving back to your own home

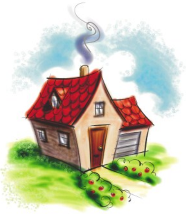

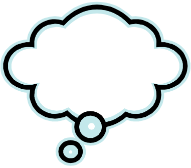

“How will I get to the chemist?”

If you find you are initially unable to get out to the chemists, don't worry, most pharmacies will deliver your medication to your house. You can phone them and request this. You can also ask your pharmacist to review your medication - they will explain what each of your tablets are for and help you if you have any questions about them. Many supermarkets also offer grocery delivery services but there is a charge for this.

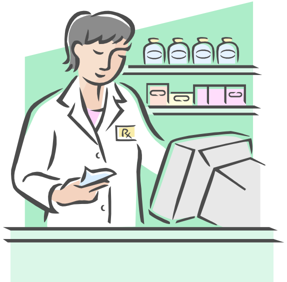

“How long until I can drive again?”

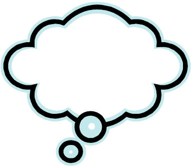

Some people may be able to return to driving eight weeks after their hip surgery, but this will depend on your recovery and it may take much longer. It is important you have regained enough strength to perform an emergency stop before you drive again - check with your healthcare professional about this and you may need to notify your insurance company about your surgery.

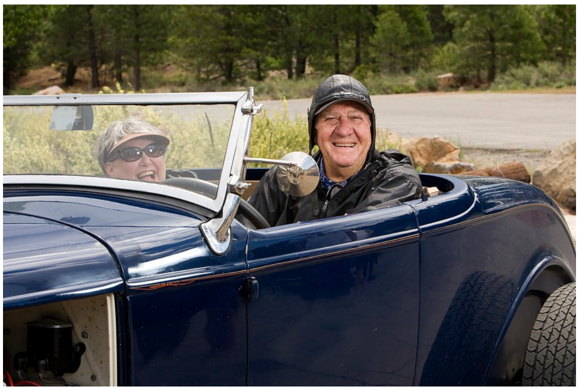

"Will I be able to get around the house like before?"

There are many different pieces of equipment that can help that are specific to your needs. For example if you are able to get around ok, but struggle with the stairs, you can have rails installed to help you balance. If you are less mobile you may need things to help with daily activities, such as a "helping hand" or "grabber" to help pick things up, or a raised toilet seat to help you go to the toilet independently. Your therapy team will discuss this with you as part of your rehabilitation and there is more information about what help may be available on page 20. There is a wide range of support available for whatever stage of recovery you are at and this can be changed as you become more active and independent.

If you move back to your own home, especially if you live alone, you may not have many people around who can give you feedback on your progress during your recovery. Make sure that you recognise your own achievements and give yourself rewards for the progress you have made and to motivate you to keep striving towards your goals!

There are also a number of charities who may be able to help support you, such as the British Red Cross Hospital at Home service, Age Cymru and others.

Moving to a community hospital

If you need more rehabilitation before going home you may go to a community hospital for a while. Community hospitals act as a bridge between the acute hospital, where you have had your surgery, and your own home. Not everybody needs this but it may be necessary if you had problems with your mobility before your fracture, or if you have other injuries or conditions that are affecting your recovery. The healthcare team will assess whether you need this extra level of care. If you are transferred to a community hospital , the amount of time you spend there can vary considerably depending on what you need. The average total length of stay in the acute and community hospitals is around 35 days for patients in Wales.

Moving to a residential or nursing home

Some people who break their hip were already finding it hard to cope at home with household tasks like cooking and cleaning, even with support from health and social services. If your mobility and ability to care for yourself is severely impaired you may require time in a residential or nursing home, or you may wish to look at sheltered housing. Although the staff will do their best to get you back to your own home, sometimes it is important to start thinking about these other care options. This is a very difficult decision, that a small number of people who fracture their hip will have to face but you will be supported by your healthcare team.

If this applies to you, the hospital and social services staff will discuss options with you and help to guide you through the process. It often takes several weeks to think this through and get things organised. While you are waiting the physiotherapists and occupational therapists will still work with you on your rehabilitation to help get you as fit and well as possible.

There is more information about how your rehabilitation will continue if you're staying in a community hospital or care home on page 21.

My hip fracture story

Anne, 65

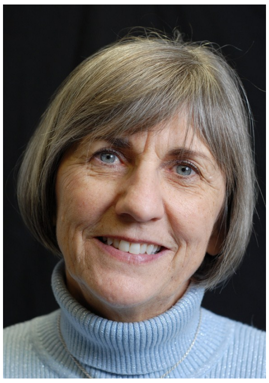

Before I broke my hip I was very active. I did a lot of gardening and liked to play golf with my friends. One day I fell on the golf course, I knew right away that I had broken my hip. I was terrified and it seemed like the ambulance took forever to arrive. At the hospital the staff were very kind and tried to make me more comfortable but I was in a lot of pain and it took three days before I was able to have my operation. After the op, the physiotherapist came to get me out of bed the next day and I was able to stand up using crutches, but I couldn't walk very well. I was in hospital for another week before they were able to send me home. I was lucky as my daughter was able to come and stay, I don't think I could have coped on my own otherwise, not right away. I also had visits from the physiotherapist to help with my exercises. It was quite a shock to feel so dependent, I thought that once the bone had healed I would be back to normal, but I felt very weak. It has taken me a lot of effort doing my exercises and a long time to recover but I hope to get back to the golf course soon!

Bryn, 82

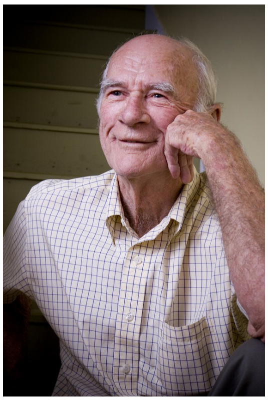

I've had arthritis for a long time, and I have problems with my chest so I struggled to get around even before my hip fracture. I live on my own and I slipped in the house one day. It was frightening and I couldn't get to the phone for a while. At the hospital they gave me pain relief but I was very confused and everything happened very quickly. I had the surgery the next day, but I didn't leave the hospital for over a week. I was still too ill to go home so they sent me to the community hospital for another five weeks. I had a lot of physiotherapy there which was very hard work and at first it didn't seem like there was any point to it, but it started to help and I could move around again if I used my frame. I was glad to be able to get to the toilet on my own. I still struggled a lot though and I couldn't cope with the stairs or cooking meals so I decided it would be better to go to a nursing home than back to my own house. It was strange at first but I like living where there are more people and I am not so scared of falling again and having nobody there to help me.

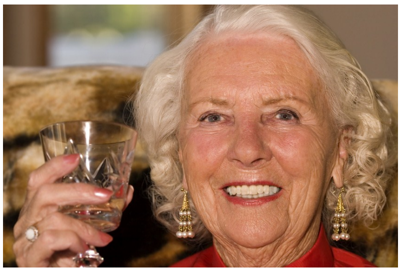

Gloria, 73

I broke my hip whilst I was out shopping with my sister, the pain was terrible. I was more scared of having the surgery though! The doctors said they would give me an epidural instead of putting me to sleep and I was worried I would be able to feel it. I had to ask a lot of questions and sometimes I felt like I was being a nuisance, but the nurses told me it was good to ask questions so I carried on! It helped me understand how it all worked and I felt much more prepared for what would happen after, so I was expecting it when they got me out of bed quite soon after the op. I was worried about going home as I don't have a lot of family close by, but another therapist came and she made sure I had everything I needed at home to help me - it was all done by the time I got taken home in the ambulance. For the first week I struggled to get in and out of bed, so I had carers who came in to help with that. I had a physiotherapist that visited me a couple of times at home to help me do exercises, but most of the time I had to practice them on my own. I was determined to get better though. I can't do all of the things I used to and I have to use a stick when I am out but I can still go and do some shopping!

My hip fracture story - Recovery

This is a space to write notes about how you are progressing with your recovery. You might find it helpful to ask your friends, family or carers about what they think as it can sometimes be difficult to recognise progress on your own.

What has been most helpful in your recovery so far? e.g exercises, equipment from the therapist, talking to others about your experience

____

____

What have you made the greatest improvement with so far?

____

____

What do you want to achieve next?

____

____

What would help you do this?

____

____

The Role of the Health & Social Care Team

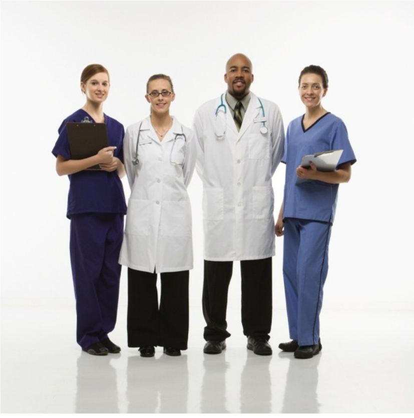

As part of your recovery and rehabilitation you will be visited by members of the healthcare team. How often you have visits and for how long will depend on where you have been discharged to and how much help you need in your recovery.

- **In your own home**

You will be given up to six weeks of care from your NHS intermediate care team and social services. The exact number of visits or appointments that you have and what will happen in them will be decided upon by your healthcare team and based on your individual needs. The physiotherapist and occupational therapist members of the team will continue your rehabilitation and monitor your progress. You will be given exercises and it is very important to carry these out as instructed. If you are able, these sessions might be carried out at physiotherapy outpatients rather than your own home. This can be useful as there will be different types of equipment that will be available to you and help with your recovery.

You may also be provided with a carer from social services who will visit to give you extra help if you need it, for example with preparing meals, going to bed or getting up in the morning. Sometimes these visits will focus specifically on helping you become more independent in these activities so you are confident carrying them out on your own.

- **In a community hospital**

If you are discharged to a community hospital before going home, you may be visited by a therapist or their assistant most days in order to help you carry out your exercises. They will give you exercises to practise everyday that you can do on your own. In addition to the exercises, an occupational therapist may also help you to plan what equipment you might need to help you when you get home.

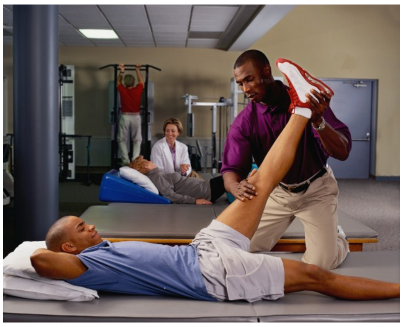

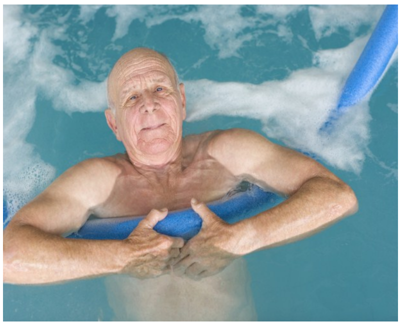

- **In a care home**

If you move into a nursing home, the nurses and carers will be mostly responsible for your care, but you will still receive visits from the physiotherapist or occupational therapist from the research team to advise you and the nurses the best way to continue with exercises or for any special equipment that may help you. You may want to ask the staff in the care home to look at the workbook and goal setting diary with you.

Because there is such variation between recovery in people who have hip fractures, the rehabilitation you are given is tailored to your needs. This means there is no "normal" package of care. Asking your healthcare team about your progress and plans is the best way to get information about your rehabilitation and how long it will take. There are also a number of useful contacts at the back of this book such as the local continence advisory service, the disability resource service and the mobility assessment service.

Quiz #2

Tick the box to indicate if you think the statement is true or false.

	True	False
There are things I can do to help my recovery	☐	☐
Most people who fracture their hip end up in a nursing home.	☐	☐
It is normal to feel frustrated or low.	☐	☐
There are lots of options for how my recovery will be structured.	☐	☐

Quiz #2: answers and discussion

"There are things I can do to help my recovery."

True - You can take control of your recovery by practising the exercises you are given and using the goal setting diary that comes with this workbook.

"Most people who fracture their hip end up in a nursing home."

False - Around half of the people who fracture their hip go straight home from the acute hospital, only a small number need more support. This may only be required in the short term.

"It is normal to feel frustrated or low."

True - Hip fracture is a major event and recovery takes time. It is natural to feel anxious about how you will cope but there is lots of help available so it is important to speak to the people involved in your care if you are worried.

"There are lots of options for how my recovery will be structured."

True - Everyone is different and will need different levels of support. Your recovery will be supported by the healthcare staff to tailor it to your needs. Speak to them to about what you want to get out of your recovery.

Recovery

Your Recovery

Setting goals

Reducing the chances of falling again

Your Recovery

After your hip has been fractured, it takes at least 12 weeks for the bone to heal. During this time your activity levels will be less than usual and this causes the muscles to weaken. As our muscles naturally weaken as we get older, this period of lowered activity is particularly problematic and makes getting back to your normal level of physical activity difficult.

The aim of your recovery is to get back to doing as much as possible of what you were able to do before your hip fracture, but this will take time and effort. Whilst it is important to stay positive and motivated about your recovery it is also important to realise that you may not recover all of the function you had before your hip fracture. It may take a year or more before you feel like you are close to being "back to normal," but some people will recover faster than this. This workbook and the treatment you receive with it is designed to help your recovery in the first 12 weeks. This will provide a good foundation for your long term recovery.

In order for your recovery to be successful you must undertake physical activities that work the muscles that have weakened. It must be strenuous enough that it builds these muscles up again. Your physiotherapist will have given you exercises that are right for you and your individual recovery. These exercises may be changed in frequency or intensity as you progress in your recovery.

Over the page there is more information about physical activity and why it is important to your recovery. Remember to take your time when starting exercise. If you can't manage an exercise or activity straight away you might be pushing yourself too hard. Follow the advice of your therapist and only do exercises that they have approved. If you have any written advice from your physio - or occupational therapist, put it in your diary so you can use it to help your recovery.

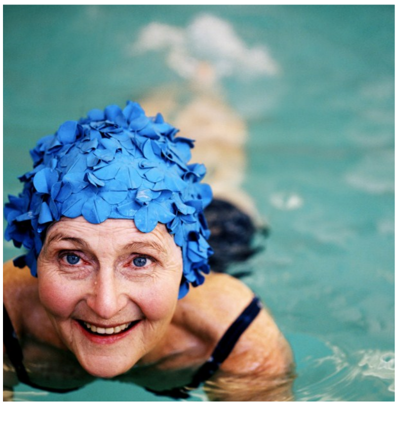

Remember when you start to increase your physical activity that:

- it will feel like hard work
- recovery can take a long time
- progress might not be obvious at first
- exercise should be gradual, frequent, comfortable and enjoyable

Stay positive when your recovery seems slow. If you are struggling with a particular activity you might want to try and do something else and come back to this at a later date when you have progressed more. Don't be discouraged - this might be a good time to look back at what you have done so far that you weren't able to do at the start of your rehabilitation, so you can see how you are getting better.

By working with your team and this workbook you can set realistic and achievable goals. When setting these goals, it is important to include some physical activities. Being physically active helps recovery because it makes your muscles and bones stronger, but it also:

- restores muscle mass
- increases fitness and keeps weight down
- improves mood and reduces stress
- improves circulation
- helps you to sleep

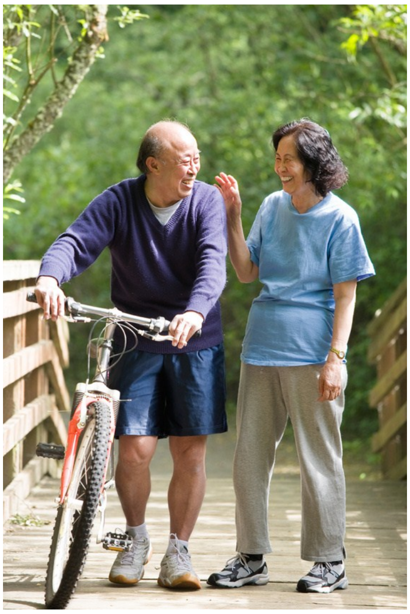

If you and your therapist feel it is a good option, you can build up your physical activity to the point where you can start to pursue more leisure- or gym-based exercise. This may be easier for some than others. Whatever your age, level of fitness, state of health it is good to think about some exercise goals.

Quiz #3

Tick the box to indicate if you think the statement is true or false.

	True	False
It takes everyone a different amount of time to recover	☐	☐
Being less active causes muscles to weaken	☐	☐
There is no need to do exercises outside of the physiotherapist visits	☐	☐
Physical activity has to be done in a gym to be effective	☐	☐

Quiz #3: answers and discussion

"It takes everyone a different amount of time to recover"

True - Recovery time depends on many factors and will be different for everyone. It is important to slowly increase the amount of exercise you do to improve your fitness but this should be done at your own pace.

"Being less active causes muscles to weaken"

True - Being less active than normal during the initial stage of recovery speeds up the natural weakening of your muscles that happens with age. This makes it harder to do your normal activities when you get back to them.

"There is no need to do exercises outside of the physiotherapist visits"

False - To get the most out of your rehabilitation you need to practice the exercises your physiotherapist has given you. Doing the right exercises is important to regain your muscle strength.

"Physical activity has to be done in a gym to be effective"

False - Not everyone will be ready to use the type of equipment available in the gym to start with. Even practicing small activities like standing up from your chair or taking a short walk will help to start with. Some light activity that is suitable for you is better than doing nothing at all.

Setting goals

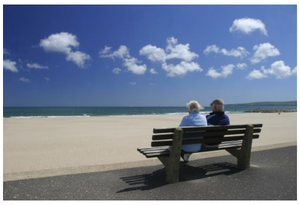

An important part of your recovery is setting goals and planning how you will achieve them. This will help you see how you are progressing in your rehabilitation. To help you succeed you should try to:

- Set time aside to think about what it is you want to achieve and how you can do this
- Plan what you are going to do
- Break the goal down into steps
- Pace yourself and readjust your goals if you're finding things too difficult
- Stay motivated and keep trying.

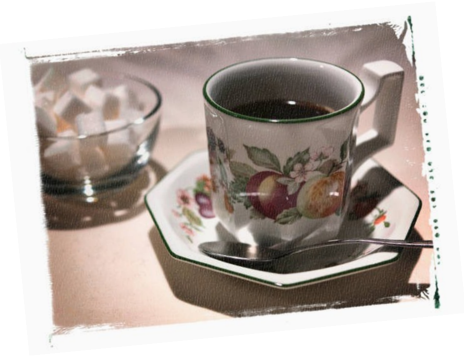

The goals that you set should push you to increase your physical activity a little at a time but should also be achievable. For some people this may be walking to the shops but for others could be using the toilet independently or making themselves a hot drink. It may be helpful to split a larger task into small steps.

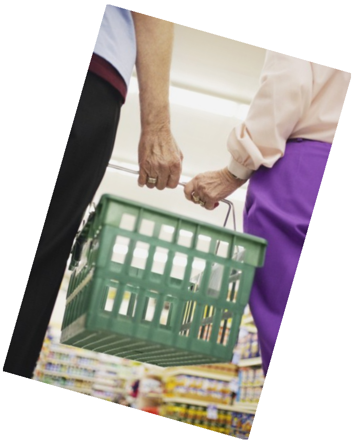

Speak with your healthcare professionals about the goals you want to set and ask them for feedback on your progress. Write them down in your goal setting diary with details of how you will achieve them. Talk through your achievements with your therapist so that you can work on things that you might be having problems with as well as enjoying your achievements. You might also want to include your family or friends when setting your goals and looking at your progress as they will be able to tell you what improvements they have seen in you.

Using the goals diary

On the next page you will see an example page of the goal setting diary. This is to help guide you in setting your own goals which should be personal to you and your recovery. Try to make them specific so you have a clear and measurable outcome. Your therapist will help you with this.

Goal setting is used by high achievers in many fields to define a long-term vision and give short-term motivation. Try and think about what you are able to do and where you would like to progress to. If you think of long-term goals you may then be able to break this down into smaller stages.

In the example, the overall goal is to walk to the shops. Some people may be able to do this sooner than others but it shows a way of breaking this down into smaller, achievable steps - aim for walking for a set time or distance each day. As this becomes easier you can increase the length of time you do it for or the distance. You may have the same overall goal from one week to the next, but the steps towards achieving it may change as your recovery progresses.

There is space to write comments on your progress. It is also important to discuss this with your therapist and get feedback.

Date __/__/__

My goal is:

To walk to the shop

I will achieve my goal by:

Walking for 5 minutes everyday

Did you work on your goal today?

	Yes	No	Comments
Mon	x		Had help from daughter
Tues		x	Too tired
Wed	x		
Thu	x		Did 8 minutes today
Fri	x		
Sat		x	
Sun		x	

How do you feel about your achievement/progress?

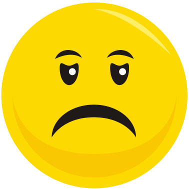

——————————X————

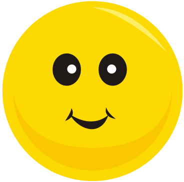

What could be done to improve how you feel about your progress?

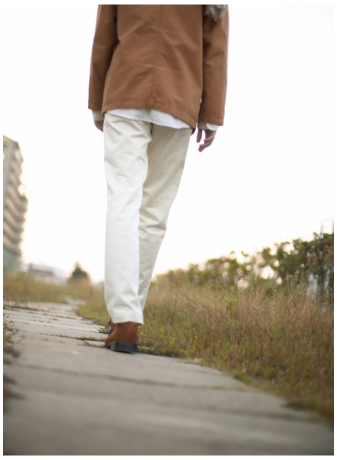

Reducing the chances of having another fall

Many people who have fractured their hip struggle to get back to their daily activities because they are frightened of falling again. This is completely normal. By practising the exercises you have been given and following the plans you have set, your strength and balance will increase and lessen your chances of falling.

There may be specific services in your area which focus on preventing falls. These may include falls prevention classes where other people who have had similar experiences will attend. Your therapist may refer you to these when they think you are ready to move on from home-based exercises. These may take place at the hospital or outpatients clinic, or as part of the National Exercise Referral scheme which is based in some leisure centres.

Falls prevention classes have a physical and emotional element. They aim to improve balance, strength, walking, co-ordination and teach techniques of how to get up if you do fall so you feel less scared and more confident. Some people who have had falls have felt that these classes were useful in their long-term recovery, and it may be particularly helpful if you feel your progress has stalled after an initial improvement. Research also shows that talking to people with similar experiences can help you feel more supported and feel less like you're alone in the challenges you may be facing.

Some people also find it reassuring to have a personal alarm service where they can call for help by pressing a button on a pendant or wristband if you fall. You may want to look into this whilst you are recovering or longer term if this works for you. Your therapist can get you more information about this and help you get this in place. The charity Age Cymru may also be able to help answer questions.

This page is review your progress in your recovery. After you have filled it in you may want to chat to your healthcare team about your answers.

How do you feel about your recovery?

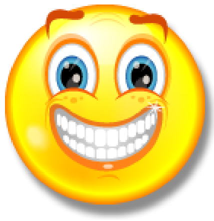

Not at all concerned

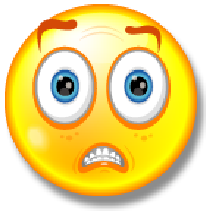

Very worried

What helps make this better?

Which exercises work best for you?

What is the biggest challenge you have overcome so far?

Is there anything else that would help improve your recovery?

Continuing your recovery

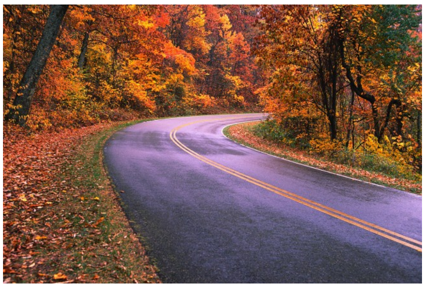

This workbook has been designed to help you with the initial part of your recovery by encouraging you to communicate with your healthcare team and helping you understand the process of your rehabilitation. Recovery can be a long road, and hopefully this workbook has helped you with the first steps on it.

It is important to build on your progress so far by continuing to set yourself realistic and achievable goals - and remember to reward yourself for the successes and hard work! Recognising your achievements and motivating yourself is an important part of your recovery.

Hopefully you are more confident in your ability to exercise and perform everyday activities safely, and with less risk of falling. Keep up the good work and try to speak to someone if you have any concerns. Talk to a family member, friend, member of your healthcare team or look in the contacts section of the workbook if you need specific advice.

Remember, stay positive and don't give up!

Workbook Progress

You can tick off which sections of the workbook you have read so that you can look back and see your progress. There is a space to note how helpful you found each of these sections - 1 is not helpful at all, 10 is very helpful

	Read	How helpful was this section?
Information about hip fracture & surgery	☐	1--2--3--4--5--6--7--8--9--10<br>Not at all Very
Information about the workbook	☐	1--2--3--4--5--6--7--8--9--10
Quizzes	☐	1--2--3--4--5--6--7--8--9--10
Hip fracture story	☐	1--2--3--4--5--6--7--8--9--10
Things to think about	☐	1--2--3--4--5--6--7--8--9--10
Leaving hospital	☐	1--2--3--4--5--6--7--8--9--10
Other peoples experiences	☐	1--2--3--4--5--6--7--8--9--10
Your health and social care team	☐	1--2--3--4--5--6--7--8--9--10
Your recovery	☐	1--2--3--4--5--6--7--8--9--10
Setting goals	☐	1--2--3--4--5--6--7--8--9--10
Using the goals diary	☐	1--2--3--4--5--6--7--8--9--10
Goal setting diary pages	☐	1--2--3--4--5--6--7--8--9--10
Reducing your chances of falling again	☐	1--2--3--4--5--6--7--8--9--10
Contact information	☐	1--2--3--4--5--6--7--8--9--10

Useful contacts

Physiotherapist

Occupational therapist

Conwy Intermediate Care Service (CICS) team

British Red Cross - Hospital at Home

Volunteers from the British Red Cross may be able to help support you in settling back into your normal routine. For more information contact your local British Red Cross branch

Bangor: 01248 364677

Abergele: 01745 828330

Holywell: 01352 718374

Age Cymru: 02920 431 555

Age Cymru are a charity who work to improve the lives of older people and help provide access to services that help people stay independent at home and doing the things they enjoy.

National Exercise Referral Scheme - Falls Intervention

National Exercise Referral Scheme - Falls Intervention provides an opportunity for patients to attend an exercise programme designed to reduce the risk of falls, led by qualified Instructors.

Elderly Accommodation Counsel (EAC): 0800 377 7070

www.housingcare.org

EAC are a charity run site providing free advice on elderly care, including specialist directories & information on nursing homes and residential care homes, as well as sheltered housing, extra care housing and retirement villages, across the UK.

Carers Wales: 0808 808 7777

A charity to support and give advice to carers in Wales

Continence advisor: 01978 725040 or 01745 443128

The Continence Advisory Service offers advice, assessment, treatment and management of bladder and bowel problems with the aim of curing or improving symptoms.

Disability Resources Centre: 01745 448288

The centre aims to provide practical services to meet the needs of disabled people and support independent living.

Wales Mobility and Driving Assessment Service: 01745 584858

Aim to provide you with long-term mobility solutions and will assess difficulties in accessing vehicles.
