# Supplementary material for: Fracture in the Elderly Multidisciplinary Rehabilitation (FEMuR): a phase II randomised feasibility study of a multidisciplinary rehabilitation package following hip fracture
Source: BMJ Open. 2016 Oct 5;6(10):e012422. doi: 10.1136/bmjopen-2016-012422 (PMC5073533; doi:10.1136/bmjopen-2016-012422)
Supplement: supplementary file [file bmjopen-2016-012422supp2.pdf]

Bwrdd Iechyd Prifysgol Betsi Cadwaladr
University Health Board

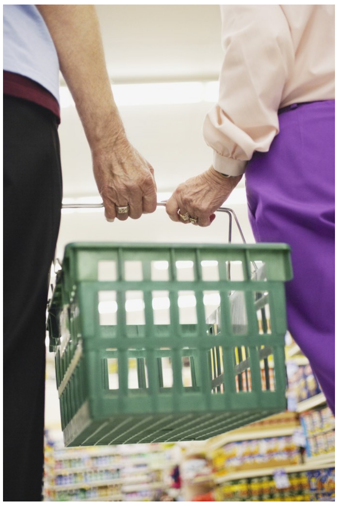

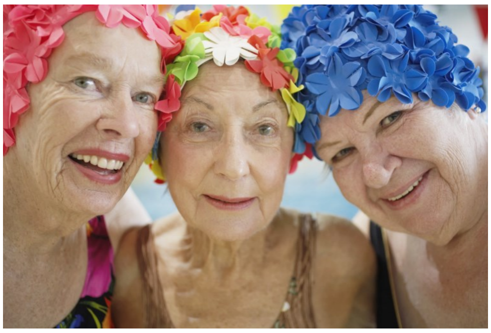

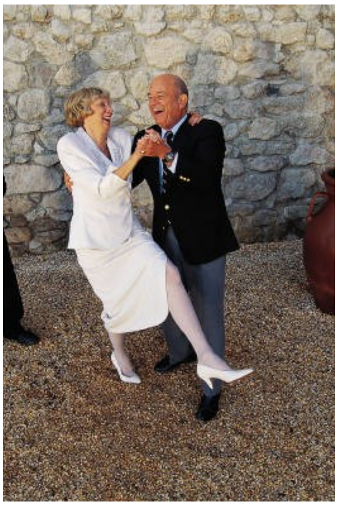

Rehabilitation Goal Setting Diary

For use with hip fracture rehabilitation workbook.

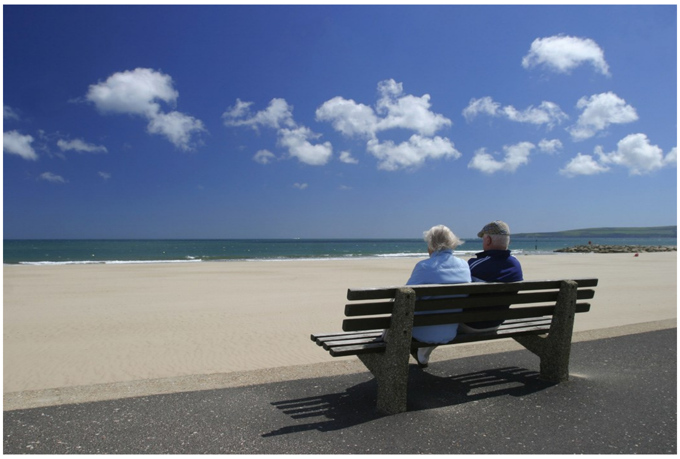

PRIFYSGOL BANGOR UNIVERSITY

NISCHR
Sefydliad Cenedlaethol ar gyfer Ymchwil Gofal Cymdeithasol ac Iechyd | National Institute for Social Care and Health Research

This diary is for setting your own personal goals for your recovery. You can use it to make a plan for how you will get back to doing things as you did before your fracture, and you will be able to look back and see your progress.

Your goals should be specific, so instead of having a goal of “getting back to normal” you might like to think about what that means to you. This will be very different for different people depending on your own health and fitness levels. To start with it is best to have short term goals that you can focus on. This might be something like being able to go to the toilet on your own, or getting out of bed without help, or making a cup of tea. In order to achieve these goals you might need to do some exercises to feel stronger, or ask your therapist for equipment that might help. These are all things that can be put in the diary to mark your progress.

When you have decided what you will doo to help achieve your goal, you may find it helpful to set an amount of time that you will do it for each day. This way you can measure how much effort you are putting in and be able to see your progress.

In time, if you are progressing well, you may want to make more long term goals, like getting back to playing golf, or going to a social event with a friend, or being discharged from the community hospital. You can break these down into smaller steps and work up to them.

Remember, progress takes time. Stay positive and ask your therapist for feedback.

Date __/__/__

My goal is:

I will achieve my goal by:

Did you work on your goal today?

	Yes	No	Comments
Mon			
Tues			
Wed			
Thu			
Fri			
Sat			
Sun			

How do you feel about your achievement/progress?

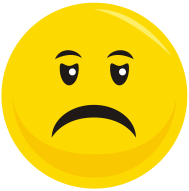
——————————————X—————
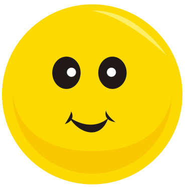

What could be done to improve how you feel about your progress?

Date __/__/__

My goal is:

I will achieve my goal by:

Did you work on your goal today?

	Yes	No	Comments
Mon			
Tues			
Wed			
Thu			
Fri			
Sat			
Sun			

How do you feel about your achievement/progress?

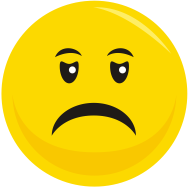 ————————————X————— 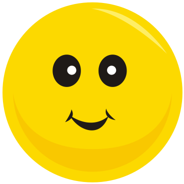

What could be done to improve how you feel about your progress?
