# Supplementary material for: Fracture in the Elderly Multidisciplinary Rehabilitation (FEMuR): a phase II randomised feasibility study of a multidisciplinary rehabilitation package following hip fracture
Source: BMJ Open. 2016 Oct 5;6(10):e012422. doi: 10.1136/bmjopen-2016-012422 (PMC5073533; doi:10.1136/bmjopen-2016-012422)
Supplement: supplementary file [file bmjopen-2016-012422supp3.pdf]

FEMuR Phase II Focus group Topic Guide – Patient/Carer

- What was your experience of the rehabilitation you received after your hip fracture?
  - What went well, what could be improved?
  - If there are any attending the focus group, what were the experiences of participants who were discharged to nursing/residential care, or those who were more dependent on carers?

Task

Rank the following aspects of the intervention according to how useful they were:

- *Extra time with a therapist*
- *Information in the workbook*
- *Being involved in setting your own goals*
- *Keeping a diary to look at progress*

Discussion of ranking

- *Did you feel confident to suggest goals to your therapists and how did that help you?*
- *What did you like best about the workbook? Was there anything you felt the workbook was missing?*
- *Were you aware of the extra sessions which you received because you were in the study?*
- *Were these sessions used differently to the care as usual sessions?*
- *Did you notice any improvements in your recovery as a result of this extra time?*

- Did your therapists give you exercises to do?
  - How confident did you feel about practising them?
  - Did your confidence change over time? What changed this?

- Often people say they were afraid of falling again after having a hip fracture, did you experience this?
  - Was there anything in your rehabilitation that helped reduce the fear of falling?
  - Did you attend a falls prevention class? How did you find out about this and was it useful?

- How did you feel about being approached to take part in the study?
  - Did you understand the information you were given?
  - What made you decide to take part?
  - How did you feel about the questions you were asked in the hospital and at the follow up, especially about how long they took and how well you were able to understand the questions and their relevance?

- Did you do some exercise tests with your community physiotherapists at the end of the study?
  - How did you feel about being asked to do these?

- How did you feel about not knowing which study group you would be in when you agreed to take part?
  - How would you have felt if you had been in the other group?

- Has anything changed for you as a consequence of being in the study?
  - If you were asked to take part now, would you make the same decision?
