# Supplementary material for: Fracture in the Elderly Multidisciplinary Rehabilitation (FEMuR): a phase II randomised feasibility study of a multidisciplinary rehabilitation package following hip fracture
Source: BMJ Open. 2016 Oct 5;6(10):e012422. doi: 10.1136/bmjopen-2016-012422 (PMC5073533; doi:10.1136/bmjopen-2016-012422)
Supplement: supplementary file [file bmjopen-2016-012422supp4.pdf]

Interview Schedule/Topic Guide: Professionals – Focus Group

- **Experiences of the enhanced rehabilitation intervention**
  What went well?
  What could be improved?

- **Experiences of extra therapist time**
  Awareness of extra time available to patients in the study
    How did if work, what went well what needs to be improved
    How did you use the extra time with patients? Do you think it made a difference?
  Views of the Workbook
    Did you use it?
    If so how, what was useful, what could be improved, was anything missing you think would have been helpful

- **Do you feel the intervention made any difference to the way patients engaged with you and participated in their rehabilitation?**
  If so how/if not why?

  What was your experience the goal setting and feedback in the work book?
    Similar/different to usual way you work
    Good points
    Areas for improvement
  What was your experience of the information sections?
    Useful to you/useful to patients/anything you weren't aware of before/ anything that was missing

  Experiences of working with patients with cognitive impairments and their carers
    How did the intervention go?

  Experiences of working with patients who were discharged to long term residential/nursing care
    How did the intervention go?

- **Feasibility of physiotherapists conducting the physical measures at 3 month follow up**

- **Acceptability of the outcome measures for patients**
  Timing
  Number
  Content

- **Anything else we haven't talked about you think has been important in working with this intervention.**

Contact: Dr Nefyn Williams Tel: 01248 382224 E-mail: nefyn.williams@bangor.ac.uk
