# Supplementary material for: Fracture in the Elderly Multidisciplinary Rehabilitation (FEMuR): a phase II randomised feasibility study of a multidisciplinary rehabilitation package following hip fracture
Source: BMJ Open. 2016 Oct 5;6(10):e012422. doi: 10.1136/bmjopen-2016-012422 (PMC5073533; doi:10.1136/bmjopen-2016-012422)
Supplement: supplementary file [file bmjopen-2016-012422supp5.pdf]

Supplementary Table 1: Reasons for readmission in cohort and feasibility study

Reasons for Readmissions	Cohort study	Feasibility study
Repeat Fall	23	0
Repeat Fracture	4	0
Other accident or injury	3	0
Adverse drug reaction	0	0
Hospital acquired infection	3	1
Revision of surgery	1	0
Cardiac/respiratory arrest	1	0
Other medical complication e.g MI, DVT, CVA, PE	28	2
Other	92	0
**Total**	**155***	**3**

*In the cohort study, there were 58 people with 1 readmission, 9 people with 2 readmissions and 2 people with 3 readmissions during the three-month follow up period. Some readmissions had multiple reasons (i.e. coded as both a repeat fall and a repeat fracture), resulting in a higher frequency of total reasons for readmission.

Supplementary Table 2: Reasons for withdrawal for patient and carer participants

Participant Type	Group	Reason for Withdrawal
Patient and associated carer	Intervention	Did not feel they could fill in goal setting paperwork.
Patient	Intervention	Too unwell with other problems
Patient and associated carer	Intervention	Burden, did not want any extra sessions
Patient and associated carer	Intervention	Burden, other co-morbidities preventing participation in therapy sessions
Patient and associated carer	Control	Were only taking part to get additional sessions
Patient and associated carer	Control	Were only taking part to get additional sessions, frustrated with lack of communication from usual health care services.
Patient	Control	Did not see the relevance of follow up questions
Carer	Control	Didn't have time to answer follow up questions
Patient	Not randomised	Decided after consent that it was too much to take on
Patient	Control	Loss of mental capacity recorded on hospital administration system at follow up, confirmed by clinical team at nursing home.

Supplementary Table 3: Completion rate for each measure collected in the trial

Measure	Baseline (61 participants, 31 carers)	Follow-Up (49 participants, 18 carers)
Outcome Measure		
Abbreviated mental test score (AMTS)	55 (90%)	Not Collected
Barthel Index	59 (97%)	44 (89%)
Carer Strain Index (CSI)	28 (90%)	18 (100%)
Client Service Receipt Inventory (CSRI)	59 (97%)*	37 (76%)
EQ5D-3L	61 (100%)	61 (100%)
Falls Efficacy Scale – International (FES-I)	Not Collected	37 (76%)
General Self Efficacy Scale (GSES)	58 (95%)	42 (69%)
Hospital Anxiety and Depression Scale (HADS)	55 (90%)	45 (92%)
Icepop Capability Measure for Older people (ICECAP-O)	58 (95%)	46 (94%)
Nottingham Extended Activities of Daily Living (NEADL)	56 (92%)	42 (69%)
Self-Efficacy for Exercise Scale (SEE)	Not Collected	36 (74%)
Visual Analogue Score – Fear of Falling (VAS-FOF)	Not Collected	47 (96%)
Visual Analogue Scale (VAS) for Hip Pain Intensity	60 (98%)	48 (79%)
Physical Function Tests		
Eight-Foot Get Up And Go Test	Not Collected	37 (76%)
Fifty Foot Walk Test	Not Collected	37 (76%)
Grip Strength	55 (90%)	31 (62%)
Thirty Second Sit To Stand	Not Collected	37 (76%)
